# Prevalence of malaria in two highly endemic Community Health Centers in the Bastar district, Chhattisgarh showing mixed infections with *Plasmodium* species

**DOI:** 10.1038/s41598-017-16974-2

**Published:** 2017-12-04

**Authors:** Sri Krishna, Ajay Yadav, Sneha Bhandari, Anup K. Vishwakarma, Praveen K. Bharti, Prem L. Mandavi, Pradeep Bahgel, Sanjay Basak, Ravendra K. Sharma, Neeru Singh

**Affiliations:** 1 National Institute for Research in Tribal Health (NIRTH), Jabalpur, Madhya Pradesh 482003 India; 2 Community Health Centre, Darbha, District Bastar, Chhattisgarh, India; 3 Community Health Centre, Kilepal, District Bastar, Chhattisgarh, India; 4 Medical Officer, District Malaria Office, Bastar, Chhattisgarh, India

## Abstract

Malaria is a major public health problem in India and in the Chhattisgarh state. The diagnosis of malaria presents a major challenge in remote areas The prevalence of malaria in Darbha and Kilepal Community Health Centers (CHCs) of the Jagdalpur district, Chhattisgarh affected by conflict was determined using microscopy and polymerase chain reaction (PCR). In the year 2015, 29.4% and 21.5% cases were found to be positive for malaria at the Darbha and Kilepal CHCs, respectively, by microscopy, and 7.4% and 1.6% of cases had mixed infections, respectively. Among the suspected cases of mixed infections and doubtful diagnoses, 21% had mixed infections with two or more species at the Darbha CHC, and 17% from the Kilepal CHC, as determined by PCR. Both the *P. vivax* subspecies Pv210 (56%) and Pv247 (44%) and the *P. ovale* curtisi subspecies were found in this area. The high proportion of mixed malaria parasitic infections detected in this study indicate the need to adequately train health staff involved in diagnosing malaria. This study showed that there is a need for site-specific data to understand the epidemiological picture and to develop appropriate intervention strategies and management guidelines for controlling and eliminating malaria in India.

## Introduction

Malaria is a major public health problem in India and contributes to 89% of malaria burden in South-East Asia^1^. A diversity of factors influences the prevalence and incidence of malaria within the malaria-endemic regions of India depending on the presence of vectors, parasite species, drug resistance, socio-economic factors, geographical areas and the types of interventions used^2,3^. According to the world malaria report (2015), a global decline in the number of malaria cases was observed from an estimated 262 million cases in 2000 to 214 million cases in 2015, which represents an approximately 18% decline. Furthermore, 57 out of 106 countries have shown a sharp reduction of approximately 75% in the incidence of malaria, and an additional 18 countries have shown an estimated 50–75% reduction in the incidence of malaria^4^.

Malaria is a major health problem in remote forested areas of the country. Recent records show that 46% of malaria, 70% of *P. falciparum* and 47% of deaths due to malaria are contributed by 7.8% of the population that resides in 124 districts of the country^5^. The diagnosis of malaria presents a major challenge in remote community health centers (CHCs) that have limited health infrastructure and human resources. In areas affected by conflict, this problem is further compounded by limited resources. Many febrile patients are still treated with antimalarial drugs without a confirmed diagnosis of malaria because the diagnosis of malaria is mainly based on microscopic examination of blood smears^6,7^. Although rapid diagnostic tests (RDTs) are easy and reliable tools for the diagnosis of *P. falciparum* and *P. vivax*, RDTs are often unable to differentiate between mixed infections. Moreover, RDTs are unable to detect a low density of parasites^7,8^. Triple and quadruple mixed malaria infections have been reported in malaria-endemic areas of Asia^9–11^ and Africa^12^. The objective of this study is to record the prevalence of malaria in two CHCs of the Jagdalpur district affected by conflict. The findings from the study will provide evidence that can be used for developing region-specific malaria control and elimination strategies in areas that are difficult to reach.

## Results

In 2015, a total of 5246 and 3253 blood smears were screened for malaria parasites using microscopy among febrile patients presenting to the malaria clinic in the CHCs of Darbha and Kilepal. Of these cases, 1541 (29.4%) and 698 (21.5%) at the Darbha and Kilepal CHCs were positive for malaria, respectively, as determined by microscopy. Malaria positivity per month varied from 16.5 to 42% (Avg SPR 29·4; 95% CI 28·1–30·6) with 83% *P. falciparum* infection at Darbha and 10.4 to 32% (Avg SPR 21.5%; 95% CI 20.1–22.9) with 90% *P. falciparum* infection at Kilepal. Malaria was recorded in all age groups ranging from 1 month to 80 years (mean age 16.12 ± 13.84). There is no difference in malaria positivity between males and females at either CHC. Age-specific and species-specific data on malaria are shown in Table 1. The slide positivity rate was lowest in adults (aged >14 yrs) when compared to all other age groups at both CHCs. At Darbha, the SVR was highest in infants (aged < 1 yrs) and showed a declining trend with increasing age (χ^2^ trend = 90.6; p < 0·0001). However, SFR was highest in relatively older children >4–8 years when compared to other age groups. A similar trend was found at the Kilepal CHC. The SVR was highest among infants and showed a linear declining trend with increasing age (χ^2^ trend = 107.5; p < 0·0001), and SFR was highest in relatively older children (>4–8 years) when compared to all other age groups. Mixed infections were 7.4% and 1.6% at the Darbha and Kilepal CHCs, respectively, as determined by microscopy. The proportion of *P. falciparum* was high (p < 0·0001) in all age groups compared to *P. vivax* at both CHCs. *P. falciparum* gametocytes were found more frequently in younger age groups (1–4 years) compared to other age groups at both CHCs.

**Table 1:** Age Group wise malaria prevalence at Darbha and Kilepal CHC (2015) of district Jagdalpur, Chhattisgarh.

Site	Age Group	BSE	Pos	Pf	Pv	Mix	PfG	SPR	SFR	SVR	PfG %	OR Malaria (95% CI)
Darbha	≤1 YEAR	346	103	51	45	7	5	29.8	16.8	13.0	8.6	1.3 (1.0–1.7)*
	>1–4 YEARS	721	260	165	66	29	19	36.1	26.9	9.2	9.8	1.8 (1.5–2.1)*
	>4–8 YEARS	565	217	160	33	24	12	38.4	32.6	5.8	6.5	1.9 (1.6–2.3)**
	>8–14 YEARS	769	269	214	38	17	8	35.0	30.0	4.9	3.5	1.7 (1.4–2.0)**
	>14 YEARS	2845	692	575	79	38	22	24.3	21.5	2.8	3.6	**Reference**
**Total**		**5246**	**1541**	**1165**	**261**	**115**	**66**	**29.4**	**24.4**	**5.0**	**5.2**	
Kilepal	≤1 YEAR	231	59	50	9	0	2	25.5	21.6	3.9	4.0	1.9 (1.4–2.6)**
	>1–4 YEARS	390	134	108	22	4	1	34.4	28.7	5.6	0.9	2.9 (2.2–3.7)**
	>4–8 YEARS	336	111	101	8	2	1	33.0	30.7	2.4	1.0	2.7 (2.1–3.5)**
	>8–14 YEARS	438	107	94	9	4	2	24.4	22.4	2.1	2.0	1.8 (1.4–2.3)**
	>14 YEARS	1858	287	266	20	1	0	15.4	14.4	1.1	0.0	**Reference**
**Total**		**3253**	**698**	**619**	**68**	**11**	**6**	**21.5**	**19.4**	**2.1**	**1.0**	

BSE: Blood slides examined; Pos: Positive for malaria; Pf: *Plasmodium falciparum*; Pv: *Plasmodium vivax*; Mix: Mixed infections; PfG: *Plasmodium falciparum* Gametocyte; SPR: Slide positivity rate; SFR: Slide falciparum rate; SVR: Slide vivax rate; PfG %: PfG Percentage.

*P < 0.05; **p < 0.001.

**Figure 1:**
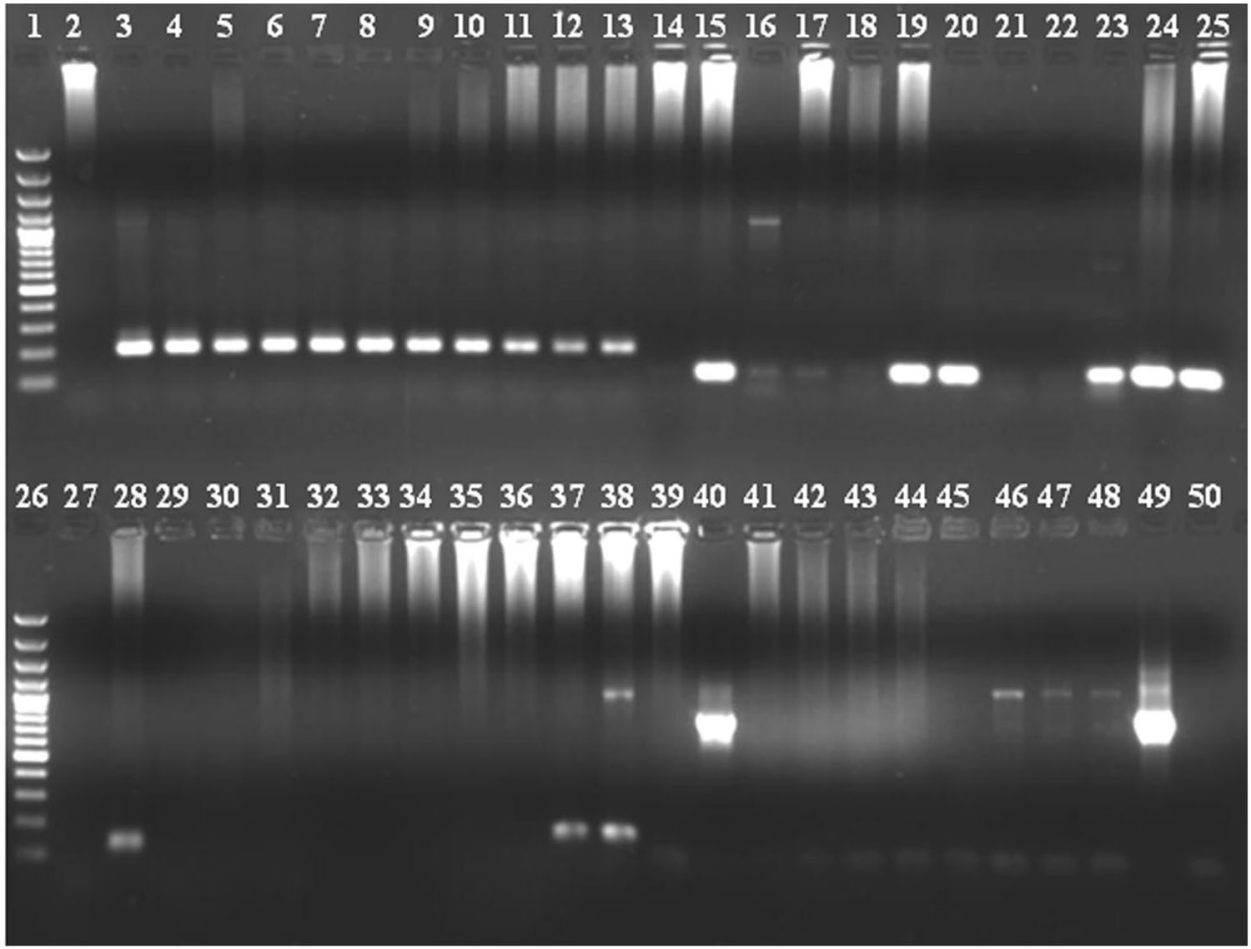
Gel image showing the results of molecular diagnosis of *Plasmodium* species by Polymerase chain reaction (PCR). **1**: 100 bp ladder; **2**: NC, **3**: PC *P. falciparum*; **4**–**13**: *P. falciparum* positive results; **14**: NC; **15**: PC *P. vivax*; **16**–**25**: *P. vivax* positive and negative results; **26**: 100 bp Ladder; **27**: NC; **28**: PC *P. malariae*; **29**–**38**: *P. malariae* positive and negative results; **39**: NC; **40**: PC *P. ovale*; **41**–**50**: *P. ovale* positive and negative results. **NC**: Negative control; **PC**: Positive control.

All available 198 blood samples with doubtful microscopic identification which were diagnosed as *P. falciparum* infection by microscopy were further confirmed by PCR. Out of 198 samples, 21% were mixed infections with two or more species at the Darbha CHC, including one case that was positive for all four species of the malaria parasite. At the Kilepal CHC, out of 157 samples tested, 17% were mixed infections with two or more species (Fig. 1). Further analysis revealed that mixed infections of *P. vivax* and *P. falciparum* were most frequent (16.7%), followed by *P. falciparum*, *P. vivax* and *P. malariae* (2.5%), *P. falciparum* and *P. malariae* (1.5%) and *P. falciparum*, *P. vivax, P. malariae* and *P. ovale* (0.51%) at the Darbha CHC (Table 2). At Kilepal, mixed infections with *P. falciparum* and *P. vivax* were also highest (16.5%), followed by *P. falciparum*, *P. vivax* and *P. ovale* (0.64%). A total of 66 mixed samples with *P. vivax* were analyzed for Pv210/Pv247 and, out of these, 56% were Pv210 and 44% were Pv247. The Pv210 was more frequently observed (62%) in the Kilepal CHC, while an almost equal proportion of Pv210 and Pv247 were found at the Darbha CHC. Both *P. ovale* samples were identified as *P. ovale* curtisi subspecies. *P*. *knowlesi* was not found at either CHC. These mixed infections were mild and did not show any complications. A significantly greater number of malaria cases (χ^2^ trend = 64.9; p < 0·0001) were recorded at Darbha compared to Kilepal, while there was no significant difference in the prevalence of mixed infection among the CHCs.

**Table 2:** Diagnostic PCR results from the Darbha and Kilepal CHCs.

CHC	Species	No of positive samples	Percentage of positive samples
DARBHA (N = 198)	Only *P.f*.	156	78.79
	*P.f*.+*P.v*.	33	16.67
	*P.f*.+*P.m*.	3	1.52
	*P.f*.+*P.v*.+*P.m*.	5	2.53
	*P.f*.+*P.v*.+*P.o*.	0	0
	*P.f*.+*P.v*.+*P.m*.+*P.o*.	1	0.51
	Negative	0	0
KILEPAL (N = 157)	Only *P.f*.	128	81.53
	*P.f*.+*P.v*.	26	16.56
	*P.f*+*P.m*.	0	0
	*P.f*.+*P.v*.+*P.m*.	0	0
	*P.f*.+*P.v*.+*P.o*.	1	0.64
	*P.f*.+*P.v*.+*P.m*.+*P.o*.	0	0
	Negative	2	1.27

## Discussion

Assessment and analysis of malaria in the local region are a prerequisite for embarking on any control and elimination program. India makes up 61% of malaria cases and 41% of malaria deaths in Southeast Asian countries^5^. Approximately 91% of India’s population lives in a malaria-affected area, while 14% of the population resides in areas where malaria transmission is high^4^. Although many cases of mixed malaria infections have been reported in malaria-endemic countries, coincidental infection with more than one species of Plasmodium is rare^9,13–16^. An earlier study from India revealed a high proportion of mixed infections (45%) with *P. vivax* and *P. falciparum* ^13^. Another study recorded 13% of mixed infections with *P. vivax* and *P. falciparum* ^17^. However, these authors did not look for *P. malariae* and *P. ovale*. Additionally, 17.4% of mixed *Plasmodium* species infections with 4 *Plasmodium* species from eight endemic states was also reported recently^9^. These studies are carried out in areas of the country where malaria is highly endemic. Mixed infections have an epidemiological significance for malaria control and elimination. For example, if *P. vivax* parasitemia is suppressed by coinfection with *P. falciparum*, effective control of *P. falciparum* infection in an area will activate *P. vivax* transmission in the community^18^, which is more difficult to control. The frequencies of less common species such as *P. malariae* and *P. ovale* are well known to be largely underestimated by microscopy^19,20^. PCR-based methods are more sensitive and more readily detect mixed species^9,13^. Using PCR, Snounou and White^21^ in Thailand found between one-third and one-half of malaria infections to be of mixed species infection. In this study, 21% and 17% of infections were found to be mixed infections at the Darbha and Kilepal CHCs, respectively. The highest number of mixed infections were found to be due to *P. falciparum* and *P. vivax* (>16%). However, we recorded only 7.4% and 1.6% of mixed infections with *P. falciparum* and *P. vivax* by microscopy at the Darbha and Kilepal CHCs, respectively.

The low sensitivity of microscopy has two major consequences in malaria control efforts. First, low-density parasitemia may serve as a reservoir for infections without the knowledge of program managers. Second, in mixed infections, the tendency of one parasite to dominate the other lowers the efficiency of microscopic detection of the two species in the same sample^22^. It is also worth mentioning that the person performing the microscopy are often inclined to identify only one species, as microscopic examination is time-consuming and labor-intensive and, as a result, the uncommon species are not detected.

The four *Plasmodium* species have varying clinical characteristics. Of the four species, *P. falciparum* causes the most severe symptoms, i.e., severe anemia, cerebral malaria, multiorgan failure and death^17,23^. *P. vivax* and *P. ovale*, though responsible for mild infections, may persist within the liver as hypnozoites, causing relapses even after treatment with blood schizonticides^18^. *P. malariae* is also mild and may persist in the human population at a very low density and may cause renal failure^24^.

The pattern of single or mixed infections is also determined by the ability of the vector species to be infected by different parasite species simultaneously^25^. In Bastar, 5 efficient vectors were found with variable prevalence and transmission potential^26–28^. It is worth mentioning that the vectors in this area transmit both subspecies (Pv210 and Pv247) of *P. vivax*, which are normally found in distinct geographically areas^29^. Although both *P. ovale* wallikeri and *P. ovale* curtisi were found in this area in a previously reported study^19^, in our current study, we only found the *P. ovale* curtisi subspecies. The quality of intervention measures, i.e., indoor residual spray coverage, distribution of bed nets, regular surveillance and drug distribution, are also very important factors and often affect transmission rates due to the remoteness and ongoing conflicts in the area^30,31^. Furthermore, health-seeking behaviours among the people, i.e., receiving treatment from quacks (unlicensed professionals) and partial treatment, are other important factors that also play a major role in maintaining reservoirs for infection.

An incorrect diagnosis of malaria is a severe public health concern, as misidentification of malaria parasites could lengthen the time to parasite clearance and can also lead to recrudescence^25^ and drug resistance^32^, especially in areas such as India where the treatment of *P. falciparum* and *P. vivax* are different. Additionally, incorrect treatment could also lead to changes in sensitivity of the parasite species to the drugs^25^. The knowledge of mixed infections is important not only for developing appropriate control measures but also for therapeutic options. This study has several limitations. Single infections or negative blood smears were not tested by PCR if parasitemia levels were too low to be detected by microscopy. Furthermore, the study was undertaken in two remote CHCs of a highly endemic district that has limited resources and transport facilities. Patients present to the CHC hospital when they are coming to the market for routine shopping. Detailed studies in different ecosystems in remote areas with larger sample sizes are required for a more accurate picture of mixed infections with common and uncommon parasite species.

In conclusion, the high proportion of mixed infections is a big challenge for the malaria elimination initiative. India launched a National Malaria Elimination Program on 10–11 February 2016 to eliminate malaria from India by 2030. The high proportion of mixed malaria parasite infections detected in this study indicates the need for adequate training of health staff involved in the diagnosis of malaria. This study showed that there is a need for site-specific data to understand the epidemiological picture for developing appropriate intervention strategies and management guidelines.

## Methods

### Study site and sample collection

**Figure 2:**
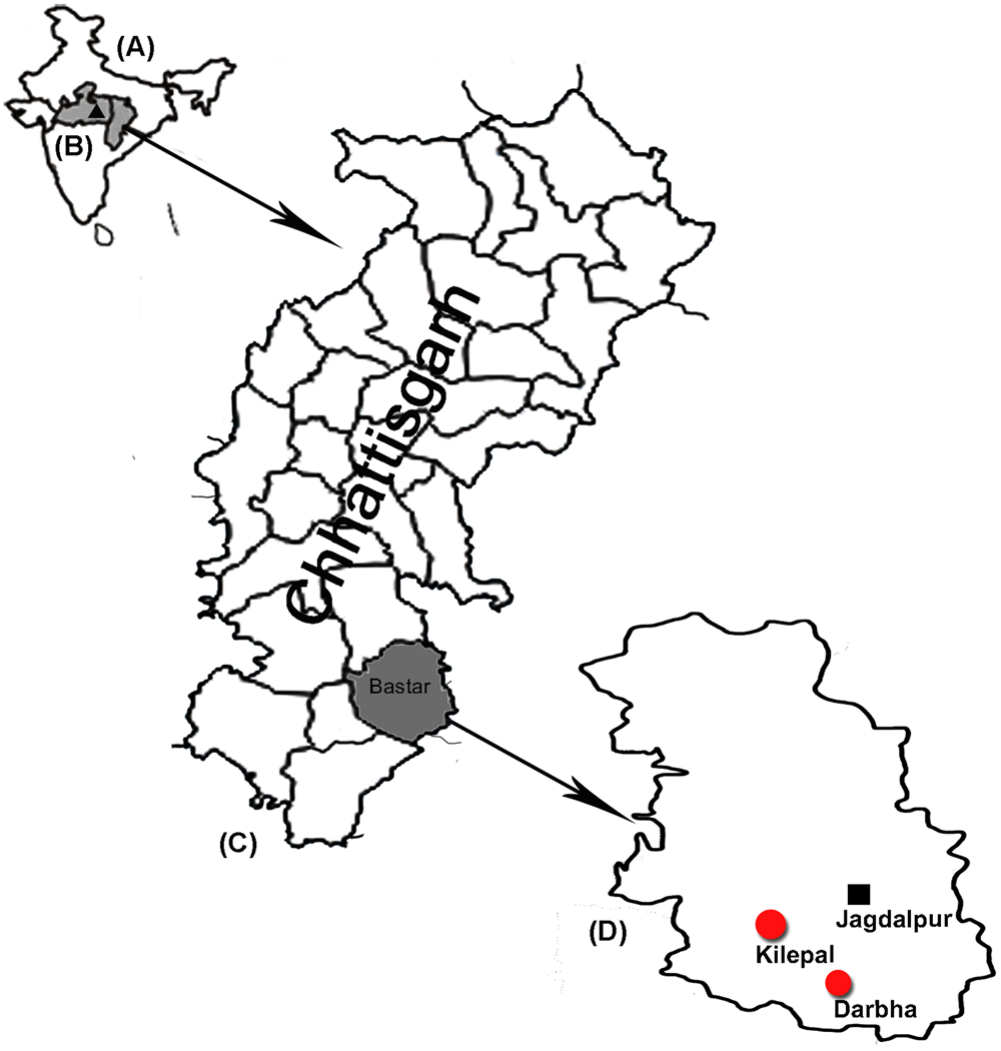
Map of India (**A**), Madhya Pradesh and Chhattisgarh (**B**), district of Chhattisgarh (**C**) and the study sites (Darbha and Kilepal CHCs) in the Bastar district (**D**). The map was generated in-house (the base map was taken from the following website: http://d-maps.com/m/asia/india/chhattisgarh/chhattisgarh66s.gif) and was modified using Adobe software Photoshop_CS3, version 10.0.

The Chhattisgarh state in Central India is the second highly malarious state in the country contributing to 14% of malaria in the country (NVBDCP - http://nvbdcp.gov.in/Doc/malaria-situation-Feb17.pdf). The Bastar district is known to have highest incidence of malaria in the Chhattisgarh state. Bastar was recently divided into seven districts, i.e., Kanker, Kondagaon, Jagdalpur, Dantewada, Bijapur, Narayanpur and Sukma^23^. The Jagdalpur district has a population of 125,463, of which 70% are of the ethnic tribe. Additionally, 50% of its geographical area is made up of forests. Jagdalpur has six community health centers (CHCs), of which two, i.e., Darbha and Kilepal, are remote community health centers (CHCs) that were selected for this study (Fig. 2). Darbha (18°51′31.0212″N81°52′7.95″E) is on the border of the Nabrangpur district of Odisha, while Kilepal (18°98′N 81°62′E) is located on the border of the Dantewada district. The villages are inaccessible due to the dense forest and valleys (temperature ranged from 22 °C to 41 °C). The population of Darbha is 79,360, and 83% of this population belong to the tribal community. The population of Kilepal is 49,334, of which 92.3% belong to the tribal community. This region is also experiencing serious problems with insurgency^33,34^, which also adversely affect health services. The economy of the villagers residing in this area is mainly forest based.

In 2015, a malaria clinic of the National Institute for Research in Tribal Health (NIRTH) of Indian Council of Medical Research (ICMR) was established at the Darbha and Kilepal CHCs (30 beds in each hospital) of the Jagdalpur district to study the prevalence of malaria in conflict-affected areas. Symptomatic patients were screened for malaria using the rapid diagnostic test (RDT SD Bioline Malaria Antigen P.f./P.v.) and with microscopy. A blood sample from a finger prick was collected from patients using sterile conditions with disposable equipment after receiving written informed consent. Thick and thin blood smears were prepared and stained with the JSB stain^35^ and were examined under the microscope. The results of the blood smears were made available in an hour. The person performing the microscopy examined 100 fields in thick smears before declaring the results negative. For quality control purposes, 100% of positive smears and 10% of negative smears were re-examined by the second expert who was unaware of the previous results. Patients were given treatment as per the National Vector Borne disease control program^36^. Patients having questionable parasite species were identified using molecular methods.

### Molecular Characterization

Genomic DNA was isolated from samples by using a commercially available FavorPrep Genomic DNA Mini Kit (Favorgen Biotech Corp., Taiwan). Diagnostic polymerase chain reaction (PCR) was carried out using a standard protocol^37^. *P. knowlesi* was also tested in all of the samples using a protocol described by Neomi *et al*. (2012)^38^.

The central repeat region of the *P. vivax* circumsporozoite protein (csp) gene was amplified and sequenced to identify the *P. vivax* subspecies (Pv210 or Pv247)^39^. A gene specific to *P. ovale* (reticulocyte binding protein gene) was also amplified to differentiate among the *P. ovale* subspecies (*P. ovale* wallikeri and *P. ovale* curtisi)^19^.

Sequencing of PCR products was conducted using the dideoxy chain termination method with forward and reverse primers using the 3730xl genetic analyzer (Applied Biosystems, USA). Sequencing results were analyzed using the sequencing analysis software v5.2 (Applied Biosystems, USA).

### Statistical Analysis

Univariate and bivariate statistical tools were used to analyze the prevalence of malaria. The odds ratios were computed to compare the slide positivity rate (SPR), slide *P. falciparum* rate (SFR) and slide *P. vivax* rate (SVR) among the different age groups. The chi-square test (χ^2^) was used to study the association of malaria prevalence among the age groups. The 95% C.I. was also computed for all univariate and bivariate analyses.

### Ethical approval

This study was approved by the institutional ethics committee of the National Institute for Research in Tribal Health (NIRTH), Jabalpur. All methods were performed in accordance with the relevant guidelines and regulations. Before collecting the samples, written informed consent was obtained from the patients or from the parents/guardians of the children as per the guidelines of the ICMR. The consent form was also provided and explained to the patients and parents/guardians of children.

### Data availability

All data generated or analyzed in this study are included in this published article.
